# A new variant of apical hypertrophic cardiomyopathy? T wave inversion and relative but not absolute apical left ventricular hypertrophy

**DOI:** 10.1186/1532-429X-15-S1-O36

**Published:** 2013-01-30

**Authors:** Andrew Flett, Viviana Maestrini, Don Milliken, Marianna Fontana, Rami Harb, Daniel Sado, Giovanni Quarta, Anna S Herrey, Perry Elliott, William J McKenna, James Moon

**Affiliations:** 1 The Heart Hospital, London, UK

## Background

**Figure 1:**
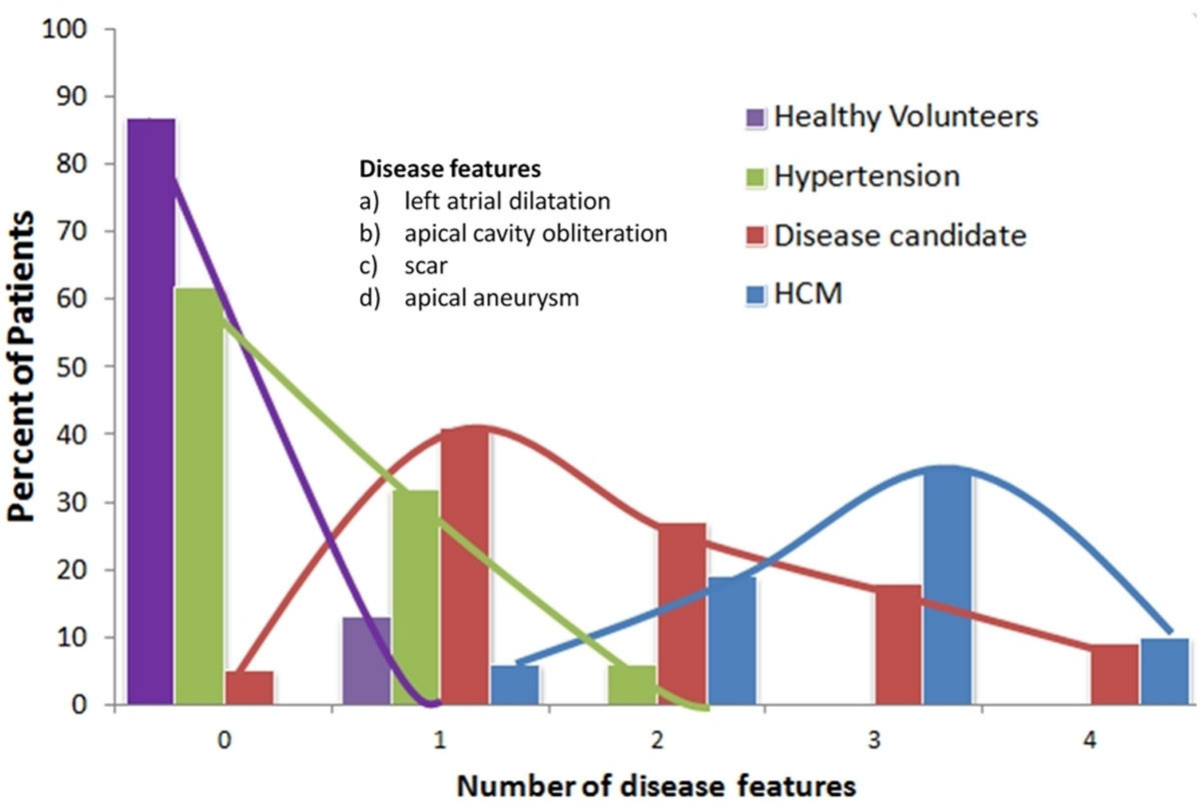
Percent of cohort with between 0 and 4 disease characteristics

Some patients present with ECG changes and symptoms that suggest HCM, but imaging using conventional wall thickness (WT) criteria is non-diagnostic. We noted that these criteria fail to take account of the normal left ventricular wall tapering towards the apex and hypothesized that there is an apical HCM variant: patients with T-wave inversion (TWI) and HCM like features with relative apical hypertrophy not fulfilling current conventional criteria (fig-1). We sought to define these characteristics compared to health, HCM and cardiac changes in hypertension.

## Methods

We retrospectively reviewed 2662 CMR scans (Siemens 1.5 T) performed over a 20 month period. Seventy-five patients were identified in whom there was TWI in the anterior leads and a suspicion of HCM. These, along with 60 healthy volunteers and 50 hypertensives, were analyzed for imaging features consistent with cardiomyopathy: relative apical hypertrophy (Apical:Basal WT Ratio: ABR>1), left atrial dilatation, >2cm apical cavity obliteration, scar and apical aneurysm.

## Results

**Figure 2:**
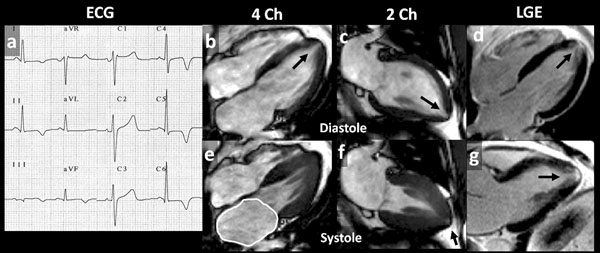
This patient with TWI has relative apical hypertrophy, left atrial dilatation, apical cavity obliteration, scar, an apical microaneurysm and yet does not fulfil current HCM diagnostic criteria

Of the 75 patients (male=56, mean 54 ± 13 years old) with TWI, 48 met conventional HCM WT criteria and went on to act as a third comparator group. Twenty seven did not meet criteria for HCM; of these 5 had no relative apical hypertrophy and were not analyzed further. The remaining 22 patients all had relative apical hypertrophy and form the candidate disease group. They also had a high rate of additional cardiomyopathy characteristics compared with controls (fig-2). Over half had 2 or more of the 4 identified features whilst no healthy volunteer had more than one, and no hypertensive had more than 2. The mean apical WT and ABR was significantly greater in the patients with TWI than in healthy volunteers and hypertensives. No healthy individual had an ABR of >0.9 and no hypertensive >0.7 i.e. the apical WT was universally thinner than the base. In fact the ABR was significantly lower in hypertension than in controls (mean 0.6±0.1mm vs 0.5±0.1mm, p<0.01). The presence of >20mm of apical obliteration was also found to be highly discriminatory with no control individual demonstrating this feature. The presence of LGE was much higher in the candidate disease group (41%) but this was also present in 8% of hypertensives.

## Conclusions

A cohort of individuals exists with T wave inversion, relative apical hypertrophy and additional imaging features of HCM that are not captured by existing criteria. We believe these represent an apical HCM variant.

## Funding

J.C.M is supported by the Higher Education Funding Council for England.

This work was undertaken at the University College London Hospital and University College London, which receive a proportion of funding from the Department of Health's National Institute for Health Research Biomedical Research Centres funding scheme.

